# High expression of 5-lipoxygenase in normal and malignant mantle zone B lymphocytes

**DOI:** 10.1186/1471-2172-10-2

**Published:** 2009-01-09

**Authors:** Yilmaz Mahshid, Marcus-René Lisy, Xiao Wang, Rainer Spanbroek, Jenny Flygare, Birger Christensson, Magnus Björkholm, Birgitta Sander, Andreas JR Habenicht, Hans-Erik Claesson

**Affiliations:** 1 Department of Medical Biochemistry and Biophysics, Karolinska Institutet, 171 77 Stockholm, Sweden; 2 Institute for Vascular Medicine, Friedrich-Schiller-University, 077 43 Jena, Germany; 3 Department of Laboratory Medicine, Division of Pathology, Karolinska University Hospital Huddinge and Institutet, 141 86 Stockholm, Sweden; 4 Department of Medicine, Division of Hematology, Karolinska University Hospital and Institutet, 171 76 Stockholm, Sweden; 5 Orexo AB, 751 05 Uppsala, Sweden

## Abstract

**Background:**

Human B lymphocytes can produce leukotriene B_4 _but the biological function of the 5-lipoxygenase (5-LO) pathway in B cells is unclear. In order to better understand and define the role of 5-LO in B cells, we investigated the expression of 5-LO mRNA and protein in subsets of B cells from human tonsils and different types of B cell lymphoma.

**Results:**

Based on RT-PCR and western blot/immunohistochemical staining, with a polyclonal antibody raised against 5-LO, high expression of 5-LO was found in mantle zone B cells from tonsils. By contrast, only a weak expression of 5-LO was detected in germinal centre cells and no expression in plasma cells from tonsils. This pattern of 5-LO expression was preserved in malignant lymphoma with high expression in mantle B cell lymphoma (MCL) and weak or no expression in follicular lymphoma. Primary leukemized MCL, so called B-prolymphocytic leukaemia cells, and MCL cell lines also expressed 5-LO and readily produced LTB_4 _after activation.

**Conclusion:**

The present report demonstrates the expression of 5-LO mainly in normal and malignant mantle zone B cells while the expression is low or absent in germinal centre B cells and plasma cells, indicating a role of the 5-LO pathway in B cells before the cells finally differentiate to plasma cells.

## Background

Arachidonic acid can be converted to leukotrienes which mediate inflammatory and immunological reactions [1]. The key enzyme in leukotriene biosynthesis is 5-lipoxygenase (5-LO), which upon activation and interaction with 5-LO activating protein (FLAP) converts arachidonic acid, via a two step process, to leukotriene (LT) A_4_. This compound can easily be transformed into LTB_4_, through the action of LTA_4 _hydrolase, or into LTC_4_, catalyzed by LTC_4 _synthase [1]. Leukotriene C_4 _can be further converted to LTD_4 _and LTE_4_. The biological effects of leukotrienes are dependent on receptor interaction [1-5]. Leukotriene B_4 _is a potent chemotactic mediator for granulocytes and T lymphocytes [6-9]. Several reports have demonstrated a function of LTB_4 _in the immune system as a stimulator of monocytes, T lymphocytes and B lymphocytes [10-12].

Biosynthesis of leukotrienes is restricted to a few cell types in the human body. Myeloid cells are the main source of leukotriene formation but B lymphocytes have also the capacity to produce LTB_4_. The activation of leukotriene synthesis in B cells is quite different in comparison to myeloid cells. Neutrophils and monocytes readily produce leukotrienes upon stimulation with calcium ionophore A23187. B cells, however, do not produce LTB_4 _after challenge with calcium ionophore only but the cells can produce similar amounts of LTB_4 _as myeloid cells after changing the cellular oxidative status [13-15]. The 5-LO activity in B cells appears to be latent and the mechanism of activation of the enzyme under physiological conditions is not yet known. Endogenously produced LTB_4_, however, plays a pivotal role in CD40-dependent activation of chronic B lymphocytic leukaemia cells (B-CLL) [16].

In resting neutrophils, 5-LO is localized in the cytoplasm but upon cell activation the enzyme translocates to the nucleus and nuclear membranes [17,18]. It has been proposed that this translocation allows for 5-LO to interact with FLAP on the nuclear membrane, thus enabling leukotriene synthesis [1]. The localization of 5-LO seems, however, to differ between different types of myeloid cells [17-19]. Phosphorylation of 5-LO appears to influence the nuclear import of 5-LO [20]. In B cell lines and isolated B cells *in vitro*, both cytoplasmic and nuclear localisation of 5-LO have been reported [14,21].

Mantle cell lymphoma (MCL) constitutes 5% of non-Hodgkin lymphomas. Most MCL carry the t(11;14)(q13;q32) translocation by which cyclin D1 becomes overexpressed [22,23]. Most MCL have unmutated immunoglobulin genes [24] and the current hypothesis is that the tumour cells are derived from the mantle or marginal zone of the B cell follicles. Microarray data of MCL have revealed high expression of 5-LO in these cells in comparison to control lymphoid tissue [25].

The enzyme 5-LO has been reported to be expressed in precursor B cells, B cell populations from the peripheral blood, tonsils and various types of malignant B cells [13,14,26]. However, it is not known which particular subsets of B lymphocytes from the tonsils which can express 5-LO and produce LTB_4_. Therefore, in order to define the function of the leukotriene pathway in B cells, we investigated the cellular expression of 5-LO in different tonsillary subsets of B lymphocytes and the corresponding type of malignant B cell lymphoma.

## Results

### PCR analysis of genes involved in the biosynthesis of leukotrienes in subsets of B cells

**Figure 1:**
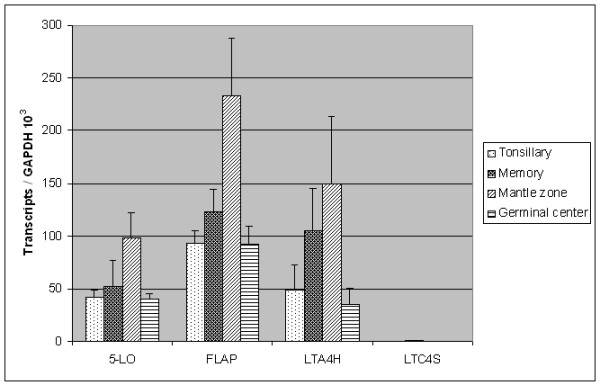
**Semiquantitative RT-PCR analysis of enzymes in the leukotriene pathway in subpopulations of B cells from tonsils**. Semiquantitative RT-PCR analysis was performed on purified non-fractioned B cells (tonsillary), memory B cells, mantle zone B cells and germinal centre B cells. The figure depicts the expression of 5-LO, FLAP, leukotriene A_4 _hydrolase (LTA4H) and leukotriene C_4 _synthase (LTC4S). Highest relative expression of 5-LO, FLAP and LTA4H was found in mantle zone B cells. The gene expression of LTC4S was absent in all the three investigated B cell subpopulations. The values are presented as "Transcripts/GAPDH 10^3 ^transcripts". Values represent experiments performed from two independent tonsils with a minimum of duplicate RT-PCR experiment per gene (mean ± SD).

RT-PCR was performed on isolated total RNA from subsets of tonsillary B cells to elucidate the gene expression of enzymes involved in the leukotriene cascade. These analyses demonstrated that 5-LO, FLAP and LTA_4 _hydrolase were expressed in various degrees in tonsillary B cells (CD19^+^), memory B cells (CD19^+^, CD38^+^, IgD^-^), mantle zone B cells (CD19^+^, CD38^+^, IgD^+^) and germinal centre (GC) B cells (CD19^+^, CD38^++^, IgD^-^). Highest relative expression of these three genes was found in mantle zone B cells and lowest expression in GC B cells (Figure 1). These results indicate differential expression of these enzymes in subpopulations of B cells. No investigated subpopulation of B cells expressed LTC_4 _synthase.

### Western blot analysis of 5-LO expression in subsets of B cells

**Figure 2:**
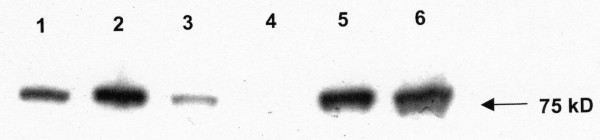
**Western blot analysis of 5-LO expression in B cells from tonsils**. Western blot was performed on different subpopulation of B cells isolated from tonsils (5 μg total protein/sample). Lane 1 – total B cells from tonsils, lane 2-mantle zone B cells, lane 3 – germinal centre B cells, lane 4 – plasma B cells, lane 5 – memory B cells and lane 6 – HL60 cells differentiated with DMSO (positive control). 5-LO protein was detected in all subpopulations except for plasma cells, with highest relative band intensity in mantle zone and memory B cells. The figure represents one typical experiment out of three independent experiments.

Subpopulations of B cells from tonsils were separated by flow cytometry and analyzed by western blot in order to further characterize the expression of 5-LO. Whole cell lysates of purified cells were submitted to SDS/PAGE followed by western blotting using a polyclonal anti-human 5-LO antibody. A single immunoreactive band at the expected size was observed in the sample consisting of total B cells (CD19^+^) from tonsils, mantle zone B cells (CD19^+^, CD38^+^, IgD^+^) and in memory B cells (CD19^+^, CD38^+^, IgD^-^) and a weak band was detected in GC B cells (CD19^+^, CD38^++^, IgD^-^) (Figure 2). In contrast, no band was detected in samples from plasma cells (CD19^+^, CD38^+++^, IgD^-^). There was a more marked difference in the expression of the 5-LO protein than in the 5-LO mRNA levels in the various subtypes of B cells (Figures 1 and 2).

### Immunofluorescence analysis of 5-LO expression in subsets of tonsillar B lymphocytes

**Figure 3:**
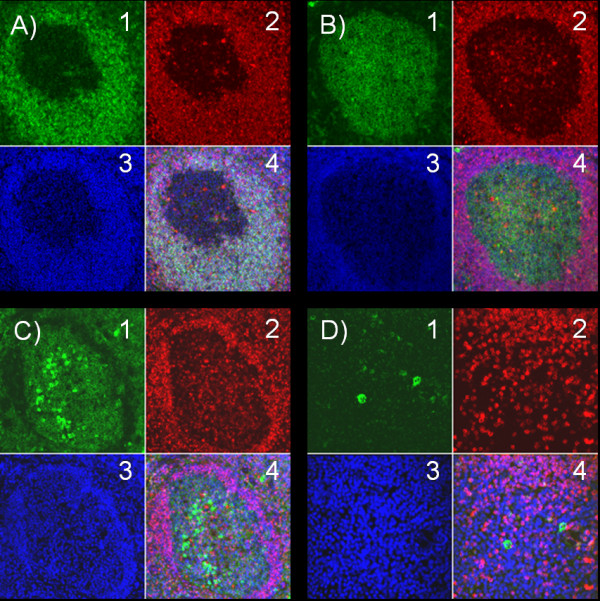
**Immunofluorescence analysis of 5-LO expression in subsets of tonsillar B lymphocytes**. Tonsils were prepared for immunofluorescence analyses and stained with anti-IgD, anti-CD38, anti-CD138 and DAPI. **A) **B cells within the mantle zone were stained with anti-IgD (green, A1); anti-5-LO (red, A2) and DAPI (blue, A3). The combined picture (A4 (A1+A2+A3)) depicts high expression of 5-LO in IgD^+ ^cells. **B) **Germinal centre B cells were stained with anti-CD38 (green, B1); anti-5-LO (red, B2) and DAPI (blue, B3). The combined picture (B4 (B1+B2+B3)) shows very weak expression of 5-LO in CD38^++ ^cells. **C) **Plasma cells within the germinal centre were stained with anti-CD38 (green, C1); anti-5-LO (red, C2) and DAPI (blue, C3). The combined picture (C4 (C1+C2+C3)) demonstrates no expression of 5-LO in CD38^+++ ^cells. **D) **Plasma cells outside the germinal centre were stained with anti-CD138 (green, D1); anti-5-LO (red, D2) and DAPI (blue, D3). The combined picture (D4 (D1+D2+D3)) depicts no expression of 5-LO in CD138^+ ^cells. The figure represents one out of four examined tonsils.

To identify the expression of 5-LO protein in subpopulations of tissue tonsillar B cells, a panel of antibodies were used to identify mantle zone B cells (IgD^+^), germinal centre B cells (CD38^++^) and plasma cells (CD38^+++ ^or CD138^+^). Figure 3A shows high expression of 5-LO in mantle zone B cells (IgD^+^). In contrast, a very weak expression of 5-LO was observed in GC cells (CD38^++^) (Figure 3B). Figure 3C demonstrates no expression of 5-LO in plasma cells (CD38^+++^) within the GC. Furthermore, no expression of 5-LO was observed in plasma cells (CD138^+^) outside the GC (Figure 3D). These results concur with the western blot analysis of 5-LO in different subsets of B cells (Figure 2).

### Immunohistochemical analysis of 5-LO expression in mantle cell lymphoma and follicular lymphoma

**Figure 4:**
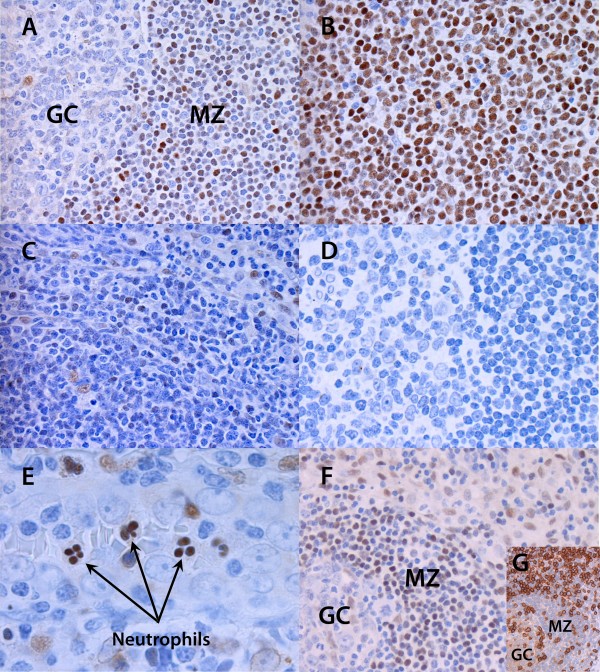
**Immunohistochemical staining of 5-LO in MCL and FL biopsies**. B cells in the mantle zone from reactive tonsils and mantle cell lymphoma express 5-LO. A) reactive lymph node, right part displays the mantle zone and the left part the germinal centre; B) mantle cell lymphoma; C) follicular lymphoma; D) negative control without primary antibody; E) nuclear staining of 5-LO in neutrophils from a normal tonsil; F) T-cells (CD3^+^) around the mantle zone are 5-LO negative, (figure G is stained with human anti-CD3 antibody). Pictures were taken with a 40×/0.85 numerical aperture (NA) and a 100×/1.30 NA objective lens. GC – germinal centre; MZ – mantle zone. The figure is a representative picture of the investigated samples listed in table 1.

In order to further characterize the expression of 5-LO in subsets of B cells, we investigated the expression of 5-LO in malignant cells derived from the mantle zone and the germinal centre i.e. mantle B cell lymphoma and follicular lymphoma. For comparison, lymph node biopsies were obtained from patients with reactive lymph nodes. In agreement with the data shown in figure 3, the expression of 5-LO in a reactive lymph node was mainly found in B cells from the mantle zone rather than from the germinal centre (Figure 4A). Interestingly, virtually all B cells expressed 5-LO in a biopsy obtained from a patient with MCL (Figure 4B). In contrast, corresponding analyses of biopsies from patients with follicular lymphoma, demonstrated that very few cells expressed 5-LO. These cells are most likely dendritic cells or tangible body macrophages as described earlier [27] (Figure 4C). No staining was observed with control antibody (Figure 4D). Similar results were obtained with another 5-LO antiserum (data not shown). Taken together, all investigated biopsies from patients with mantle zone B cell lymphoma clearly expressed 5-LO, whereas, little or none staining was observed in biopsies from patients with follicular lymphoma (Table 1).

**Table 1:** Expression of 5-lipoxygenase in reactive lymphoid tissue, mantle cell lymphoma and follicular lymphoma

**Patient number**	**Age**	**Sex**	**Tumor**	**Subtype**	**5-LO Expression**
1	56	F	Lymph node	N.A.	moderate
2	19	F	Lymph node	N.A.	moderate
3	43	F	Tonsil	N.A.	moderate

4	90	M	MCL	classic	moderate
5	83	M	MCL	classic	strong
6	56	M	MCL	classic	strong
7	82	F	MCL	classic	moderate
8	81	F	MCL	classic	weak
9	77	M	MCL	classic	strong
10	81	M	MCL	classic	strong
11	49	M	MCL	classic	strong
12	63	F	MCL	blastoid	weak
13	68	M	MCL	blastoid	moderate

14	37	M	FL	FL1	weak
15	70	F	FL	FL1	weak
16	34	F	FL	FL1	negative
17	73	M	FL	FL2	negative
18	40	F	FL	FL2	negative
19	73	F	FL	FL2	negative
20	58	M	FL	FL2	negative
21	61	M	FL	FL3a	negative
22	66	M	FL	FL3a	negative
23	49	F	FL	FL3a	negative

5-LO protein expression was evaluated by immunohistochemistry. Samples of reactive lymphoid tissue (lymph nodes, (n = 2) and tonsil, (n = 1)), mantle cell lymphoma (MCL), classic and blastoid variants (n = 10) and follicular lymphoma (FL), malignancy grades 1-3a (n = 10) were collected in a tissue microarray and simultaneously stained. The age and the sex of the patients are indicated. Expression of 5-LO in lymphoma cells was semiquantified in four levels; absence of staining in tumor cells (negative), weak staining (weak), moderate staining (moderate) and strong staining (strong) as compared to cells in reactive mantle zones that were moderately positive. N.A. = not applicable

As a comparison, tonsillar polymorphonuclear granulocytes expressed 5-LO in the nucleus (Figure 4E), in contrast to tonsillar CD3 positive T lymphocytes which did not express 5-LO (Figures 4F and 4G). The IHC in figure 4G was stained with human anti-CD3 antibody.

### Biosynthesis of LTB_4_ in mantle B cell lymphoma

**Figure 5:**
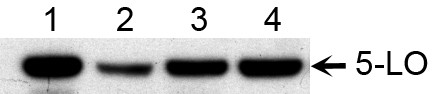
**Western blot analysis of 5-LO in three MCL cell lines**. Western blot was performed on three different MCL cell lines (Granta 519, JEKO-1 and Rec1) with an anti-human 5-LO antibody. Lane 1 – PMNL (positive control), lane 2 – Granta 519, lane 3 – JEKO-1 and lane 4 – Rec1. An aliquot (7 μg) of total protein was analysed for each sample. The figure represents one experiment out of three independent experiments.

**Figure 6:**
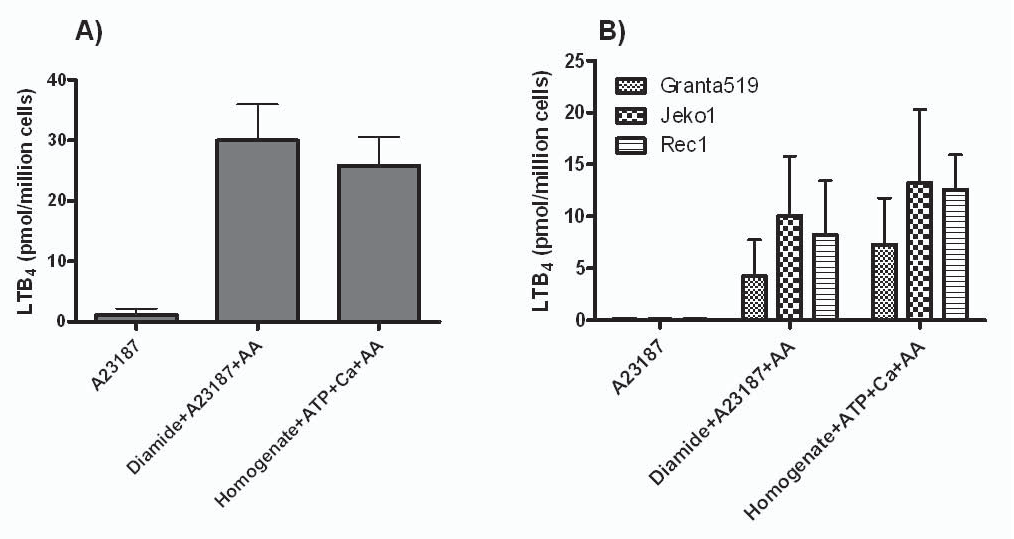
**Biosynthesis of LTB_4 _by B-PLL cells and MCL lines**. Intact cells (10 × 10^6^) were pre-incubated for two min at 37°C, in the presence of Diamide (100 μM), prior to addition of A23187 (1 μM) plus arachidonic acid (AA) (40 μM) for five min. Sonicated cells (homogenate) were pre-incubated with ATP (1 mM) for two min at 37°C and then incubated with calcium chloride (2 mM) and arachidonic acid (40 μM) for five min. Panel A depicts the formation of LTB_4 _in B-PLL cells isolated from four different donors (mean ± SD). Panel B depicts the formation of LTB_4 _in three different MCL lines i.e. Granta 519, JEKO-1 and Rec1 (mean ± SD, from three independent experiments).

Since it was difficult to isolate large numbers of tonsillary non-malignant mantle zone B cells for chemical analysis, we used MCL cells to study the biosynthesis of LTB_4 _in mantle zone B cells. For this purpose, we used both different MCL cell lines and primary MCL cells isolated from the peripheral blood of patients with leukemized MCL, so called prolymphocytic leukemia (B-PLL) with t(11;14). B-PLL is in fact a heterogenous disease and those cases that are positive for cyclin D1 and carry the t(11:14) translocation are in the current WHO classification of malignant lymphomas [22] considered to be leukemic forms of MCL [28,29]. All three investigated MCL cell lines (Granta 519, JEKO-1 and Rec1) expressed 5-LO protein (Figure 5). Figure 6 depicts the capacity of B-PLL cells and MCL cell lines to produce leukotrienes. The challenge of B-PLL cells with ionophore A23187 plus arachidonic acid and the thiol-active compound Diamide led to the formation of similar amounts of LTB_4 _as produced by human neutrophils (Figure 6A) [30]. This cocktail of compounds is known to induce LTB_4 _production in other types of B lymphocytes [14]. Sonicated cells also readily produced LTB_4 _(Figure 6A). Upon calcium ionophore activation only, isolated B-PLL produced low amounts of LTB_4 _(about 1 pmol/10^6 ^cells), showing that there were minimal amounts of myeloid cells contaminating these preparations of B-PLL cells. Figure 6B shows that intact or sonicated Granta 519, JEKO-1 and Rec1 cells also produced significant amounts of LTB_4 _as B-PLL cells. The capacity of the different cell lines to produce LTB_4 _correlated relatively well with the degree of expression of 5-LO protein (Figures 5 and 6B). In essence, these results demonstrate that both B-PLL cells and MCL cell lines have the capacity to produce LTB_4 _and that the cells contained substantial amounts of 5-LO, which could be activated under certain conditions.

## Discussion

The activation mechanisms and biological functions of 5-LO in human B lymphocytes are unclear although the overall capacity of B cells to produce LTB_4 _is similar to myeloid cells [30]. We purified various subpopulations of tonsillary B cells and investigated the expression of enzymes involved in the leukotriene pathway. RT-PCR and western blot analysis of B cells from tonsils separated by flow cytometry showed high expression of 5-LO, in particular mantle zone B cells but also memory B cells (Figures 1 and 2), while germinal centre cells and plasma cells showed low or no expression of 5-LO, respectively. This analysis also demonstrated that FLAP and LTA_4 _hydrolase were expressed in these various B cell populations (Figure 1). In order to further investigate the expression of the 5-LO protein, immunofluorescence analyses of tonsillary tissue B cells were performed. A distinct expression of 5-LO was seen in IgD^+ ^B cells in the mantle zone, while only a weak expression was found in germinal centre B cells and virtually no expression in plasma cells (Figure 3).

To verify if the expression of 5-LO was preserved in malignant B cells we investigated MCL and FL. Mantle cell lymphomas have a lower somatic mutation rate in immunoglobulin genes than follicular lymphomas and approximately 60–75% of mantle cell lymphomas have immunoglobulin genes in germ-line configuration. Therefore, it has been hypothesized that mantle cell lymphomas are derived from naïve cells, similar to normal mantle zone B cells [31]. Immunohistochemical analysis of biopsies from MCL patients showed high expression of 5-LO in virtually all cells (Figure 4B). Follicular lymphomas, on the other hand, have a high rate of somatic mutations in immunoglobulin genes and clearly originate from germinal centre B cells [31]. There were no or low expression of 5-LO in follicular lymphoma cells and this concur with the results of normal germinal centre B cells (Figures 2, 3, 4A, 4C and Table 1).

There are conflicting data about the expression of 5-LO in T cells. We have previously reported that T cells express FLAP but not 5-LO [13], whereas, one other report indicates the formation of LTB_4 _by human T cells [32]. We found no evidence for the expression of 5-LO protein in CD3 positive tonsillar T cells (Figure 4F).

Since the numbers of tonsillary mantle zone B cells that can be obtained by flow cytometry cell sorting is limited, we investigated the capacity to produce LTB_4 _in primary B-PLL cells and cell lines derived from patients with MCL. Three different MCL cell lines were used in this study and all were found to express 5-LO and to have the capacity to produce LTB_4 _(Figures 5 and 6). Also, primary B-PLL cells produced LTB_4 _in amounts similar to that of myeloid cells. Primary MCL/B-PLL cells and MCL cell lines produced LTB_4 _after being challenged with calcium ionophore plus arachidonic acid and Diamide but not after stimulation with calcium ionophore only, showing that the mechanism of activation of the leukotriene pathway in MCL cells is similar to other investigated B cells [16,26,33].

This study and earlier reports demonstrate that 5-LO is expressed in peripheral B cells, IgD^+ ^B cells in the mantle zone, memory B cells and in various malignant B cells, e.g. CD10^+ ^acute pre-B-lymphocytic cells, chronic B-lymphocytic leukaemia, B-PLL and MCL [13,14,26]. Investigated B lymphocytes that do not express 5-LO are germinal centre derived B cells, plasma cells and CD10^- ^acute pre-B-lymphocytic cells. The question if and under which conditions B lymphocytes can release LTB_4 _*in vivo*, is still unclear. In fact, it is possible that the role of the 5-LO pathway is quite different in B cells than in myeloid cells, and that the pathway has only an endogenous function in B cells which do not release and export LTB_4_. Thus, in B cells, 5-LO and LTB_4 _might only have a role at the level of the nucleus. However, the physiological conditions required for activation of the 5-LO pathway in B cells and biosynthesis of LTB_4 _have not yet been uncovered. In this regard, the cellular oxidative status of the cells seems to be of importance [13-15]. Our finding that the enzyme is highly expressed in mantle zone B cells and less expressed in germinal centre B cells and not expressed in plasma cells could indicate that the enzyme might be activated in the lymph node to attract activated T cells. It is known that activated T cells express BLT1, the LTB_4 _receptor, and that LTB_4 _is a potent chemotactic agent for T cells [7-9]. Therefore, we will now study the effect of T- and B lymphocyte interaction on the activity and expression of 5-LO.

## Conclusion

In summary, the present study demonstrates high expression of 5-LO mantle zone B cells but not in germinal centre B cells or plasma cells indicating a role of the 5-LO pathway in B cells before the cells differentiate to plasma cells.

## Methods

### Reagents and cell lines

The calcium ionophore A23187 was purchased from Calbiochem-Behring (La Jolla, CA, USA). HPLC solvents were obtained from Rathburn chemicals (Walkerburn, U.K.) and the synthetic standards of LTB_4 _and prostaglandin (PG) B_1 _were from Biomol (Plymouth, PA, USA). Azodicarboxylic acid bisdimethylamide (Diamide) was from Sigma-Aldrich (Stockholm, Sweden) and arachidonic acid (AA) from NU-CHEK PREP Inc. (MN, USA). The well-characterized MCL cell lines Granta 519 and JEKO-1 were purchased from Deutsche Sammlung von Microorganismen und Zellkulturen (DSMZ) (Braunschweig, Germany). The MCL cell line Rec1 [34] was a generous gift from Dr. Christian Bastard (Ronan, France). Cell lines were maintained in RPMI 1640 medium (Invitrogen Life Technologies, Stockholm, Sweden) supplemented with 2 mM L-glutamine and 10% fetal calf serum (FCS) and 50 μg/mL gentamicin (Invitrogen) under standard conditions (humidified atmosphere, 95% air, 5% CO_2_, 37°C). JEKO-1 was maintained as described above but with a final concentration of 20% FCS.

### Isolation of subpopulations of B cells

Tonsils were obtained from patients undergoing tonsillectomies (HELIOS Klinikum, Erfurt, Germany). Isolated cells from tonsils were incubated with CD19 micro beads (Miltenyi, Germany) and loaded in a magnetic field on a MACS separation column LS (Miltenyi). Cells were washed three times with PBS/EDTA (2 mM)/FCS (5%), subsequently the column was removed from the magnetic field and CD19 positive cells were eluted with 4–6 mL PBS/EDTA/FCS. Isolated cells were concentrated to 10^7 ^cells/50 μL and incubated with anti-CD38-PE (Pharmingen, Germany) and anti-IgD-FITC (Pharmingen) for 20 min at 4°C. Cells were washed twice, set to 2 × 10^6 ^cell/mL in PBS/EDTA/FCS and sorted with a FACSVantage SE cell sorting instrument (Beckton Dickinson, Germany). B cells were classified according to the scheme described by Grammer et al. 1999 [35], mantle zone B cells (CD19^+^, IgD^+^, CD38^+^), germinal centre B cells (CD19^+^, IgD^-^, CD38^++^), memory B cells (CD19^+^, IgD^-^, CD38^+^) and plasma cells (CD19^+^, IgD^-^, CD38^+++^). Only cell populations that were > 95% pure were used in further experiments.

### Isolation of total RNA and RT-PCR

Total RNA was separated according to the manufacturer's protocol with an RNeasy mini kit (Qiagen, Germany). Concentration and quality of RNA was determined with a Bioanalyzer2100 (Agilent, Germany). The reversed transcription reaction was performed on 1.8 μg of total RNA in a RT-PCR mixture (1× RT buffer, 167 μM dNTP, 0.7 U/μL RNasin, 0.1 μg/μL BSA, 0.35 U/μL AMV reverse transcriptase (Roche, Germany)).

### PCR

PCR was performed with 60 ng of cDNA for each reaction. The PCR reaction mixture contained; PCR buffer, dNTP mix (0.2 mM each), BSA (0.1 μg/μL), SybrGreen (1:50) (Roche), MgCl_2 _(1.8 mM), Platinum Taq DNA polymerase (1.5 U) (Invitrogen, Germany) and primer mix (0.2 μM each). Primers that were used are described in table 2.

**Table 2:** List of primers that were used in the RT-PCR experiment

**Gene**	**Annealing temp.**	**Size of PCR Product**	**sequence**
5-LO	72°C	486 bp	5'ACCATTGAGCAGATCGTGGACACGC 3'GCAGTCCTGCTCTGTGTAGAATGGG

FLAP	71°C	352 bp	5'GGCCATCGTCACCCTCATCAGCG 3'GCCAGCAACGGACATGAGGAACAGG

LTA4H	72°C	464 bp	5'GCAGTCACGGGATGCATGCTTGCT 3'GCCTGGCTCTACTCTCCTGGACTG

LTC4S	68°C	252 bp	5'TGCCACCACACCGACGGTACCATG 3'CCCTTCATGAAAGAAGATGCCGG

### Western blot

Tonsillary B cell samples were washed in PBS (2×) and re-suspended to 4 × 10^4 ^cells/μL in 5× sample buffer (250 mM Tris/HCl pH 6.8, 30% Glycerol (v/v), 10% SDS (w/v), 5% β-mercaptoethanol (v/v), 0.02% Bromphenolblue (w/v), complete mini protease inhibitor (Roche)). The suspension was heated to 95°C (5–10 min) and run on a regular 10% SDS-PAGE with SDS running buffer (25 mM Tris/HCl; 190 mM Glycine; 0.1% SDS (w/v)). HybondC nitrocellulose membrane (GE Healthcare, Germany) and semi-dry blotting apparatus (LMS labortechnik, Germany) was used for western blot transfer. The detection was performed with ECL kit (GE Healthcare, Germany). Primary antibody used for the immunoblot detection was anti-5-LO polyclonal rabbit antiserum [36] and secondary antibody was an anti-rabbit-IgG coupled with HRP (GE Healthcare, Germany).

MCL cell lines were washed in PBS (w/o calcium and magnesium) and suspended in PBS (w/o calcium and magnesium) supplemented with complete mini protease inhibitor (Roche, Sweden). Cells were sonicated on ice (3 × 5 seconds) (Sonics Vibra cell, 40% amplitude, CiAB, Sweden) and centrifuged (1 × 10^5 ^× g, 60 min, 4°C). Supernatant was collected and protein concentration was determined with Bradford. Protein (7 μg) was loaded and separated on a 7% tris-acetate gel. PVDF membrane (GE Healthcare, Sweden) and semi-dry blotting apparatus (GE Healthcare, Sweden) was used in the western blot transfer.

### Immunofluorescence analysis of tonsillary B cells

Confocal laser scan microscopy was performed with 7 μm cryostat sections embedded in Tissue Tec (Sakura, Japan). Specimens were placed on glass slides and fixed with acetone for 15 min at 4°C. After rehydration in PBS supplemented with BSA (2%) for 30 min at 20°C, specimens were incubated for 1 h at room temperature with rabbit anti-5-LO antiserum and for double immunofluorescence analyses with unlabeled primary antibodies against IgD and CD38 (Pharmingen), CD19 (Coulter Immunotec) or CD138 (Serotec). Nuclei were counterstained with DAPI. Secondary antibodies (goat anti-mouse-IgG F(ab')2-Cy2 and donkey anti-rabbit-IgG F(ab')2-Cy3; Dianova) were applied for 1 h at room temperature. Sheets were mounted on coverslips with Permafluor (Beckman, Munich, Germany) and viewed on a Zeiss Axiovert 200 M microscope equipped with a confocal laser scanning head (LSM510). Pictures were taken and analyzed using LSM510 Image Examiner software (Zeiss, Jena, Germany).

### Immunohistochemistry on Tissue Microarray (TMA)

Formalin-fixed, paraffin-embedded tumor (lymph nodes) tissue samples and hyperplastic tonsils were obtained from the Department of Pathology, Karolinska University Hospital Huddinge. Before constructing a TMA block, serial 5 μm sections were cut from each donor block. One of these sections was stained with H&E for marking morphologically representative areas of the tumor. One representative area was targeted. Using Beecher Instruments Tissue Arrayer (Silver Springs, MD, USA), tissue cylinders with a diameter of 1.0 mm were punched from the targeted area in each donor block and deposited into a recipient TMA block. The cores of each TMA included duplicate cores from all cases and two cores of normal human heart muscles as a control for orientation of the sections.

Multiple serial 5 μm-thick TMA sections containing samples were cut de-paraffinized in xylene and hydrated in a series of graded alcohols. Enzyme digested antigen retrieval was carried out with Protease (Sigma P-5147, Sweden) at 20°C for 10 min. Anti-5-LO antibody (LO-32, Merck Frosst, Canada) was used at a dilution of 1:300 and anti-CD3 antibody was used at a dilution of 1:100 (DAKO). All staining were semiautomatic and performed on a TechMate 500 plus (DAKO, Glostrup, Denmark) by using the Dako REAL™ Detection System, PeroxidaseDAB, RabbitMouse kit as recommended by the manufacturer.

The results of the staining were evaluated by three persons (XW, BC and BS). Images of immunohistologic staining were acquired using an Olympus BX45 microscope (Olympus, Stockholm, Sweden) and Sony digital camera (DXC-S500, Sony, Stockholm, Sweden). Digitized images were processed using Picsara 8.9 Rev 5 software (Bildanalyssytem AB, Stockholm, Sweden).

### Incubation of B-PLL cells and MCL cell lines

Intact cells (10 × 10^6^) were suspended in 1 mL phosphate buffered saline (PBS) and pre-incubated for two min with azodicarboxylic acid bisdimethylamide (Diamide) (100 μM) prior to stimulation with arachidonic acid (40 μM) and calcium ionophore A23187 (1 μM). The cells were stimulated for five min and the reaction was terminated with 1 mL methanol. The A23187 incubation was performed as described above but without any addition of Diamide and arachidonic acid. Alternatively, cells (10 × 10^6^) were re-suspended in 1 mL calcium free PBS including EDTA (2 mM) and sonicated for 3 × 5 seconds. The cells were pre-incubated for two min at 37°C in the presence of ATP (1 mM) prior addition of calcium chloride (2 mM) and arachidonic acid (40 μM). The reaction was terminated after five min of incubation with 1 mL methanol.

### Analysis of leukotrienes

After addition of PBS (0.5 mL) and the internal standard PGB_1 _(50 pmol), the samples were centrifuged (1250 × g, 5 min). The supernatants were subsequently subjected to solid phase extraction on Oasis HLB Extraction Cartridges (10 mg, Waters, Sweden). The methanol fractions were analyzed on a Waters Alliance 2695 reverse phase HPLC and detected with Waters PDA 996. Methanol:water:trifluoroacetic acid (70:30:0.007, v/v) was used as mobile phase at a flow rate of 1.2 mL/min. Qualitative analysis was performed by comparison of retention times of synthetic standards and quantitative determinations were performed by computerized integration of the area of eluted peaks. Mean and standard deviation was calculated.

### Ethical perspective

This study was approved by the local ethic committee of Friedrich-Schiller-University of Jena, Germany, and Karolinska University Hospital, Stockholm, Sweden.

## Abbreviations

5-LO: 5-lipoxygenase; AA: Arachidonic acid; B-PLL: B-Prolymphocytic leukaemia; Diamide: Azodicarboxylic acid bisdimethylamide; FL: Follicular lymphoma; FLAP: 5-LO activating protein; GC: Germinal centre; LT: Leukotriene; MCL: Mantle cell lymphoma; MZ: Mantle zone; TMA: Tissue microarray.

## Authors' contributions

YM carried out the 5-LO activity experiments and western blot, participated in designing the experiments and drafted the manuscript. MRL performed RT-PCR, western blot and immunofluorescence experiments, participated in designing the experiments and helped to form the final manuscript. XW performed the TMA experiments, participated in designing experiments and helped to draft the manuscript. RS participated in the design and evaluation of the experiments and helped to form the final manuscript. JF participated in designing and evaluation of the experiments. BC participated in designing and evaluation of the experiments. MB participated in the evaluation of the experiments and helped to form the final manuscript. BS participated in designing and evaluation of the experiments and wrote parts of the manuscript. AJRH conceived of part of the study, and participated in its design and helped to draft the manuscript. HEC conceived of part of the study, and participated in its design and drafted the manuscript. All authors have read and approved the final manuscript.
